# Simultaneous Optimization of Charge Transport Properties in a Triple-Cation Perovskite Layer and Triple-Cation Perovskite/Spiro-OMeTAD Interface by Dual Passivation

**DOI:** 10.1021/acsomega.2c01195

**Published:** 2022-05-17

**Authors:** Adem Mutlu, Tamer Yeşil, Deniz Kıymaz, Ceylan Zafer

**Affiliations:** Solar Energy Institute, Ege University, 35100 Izmir, Turkey

## Abstract

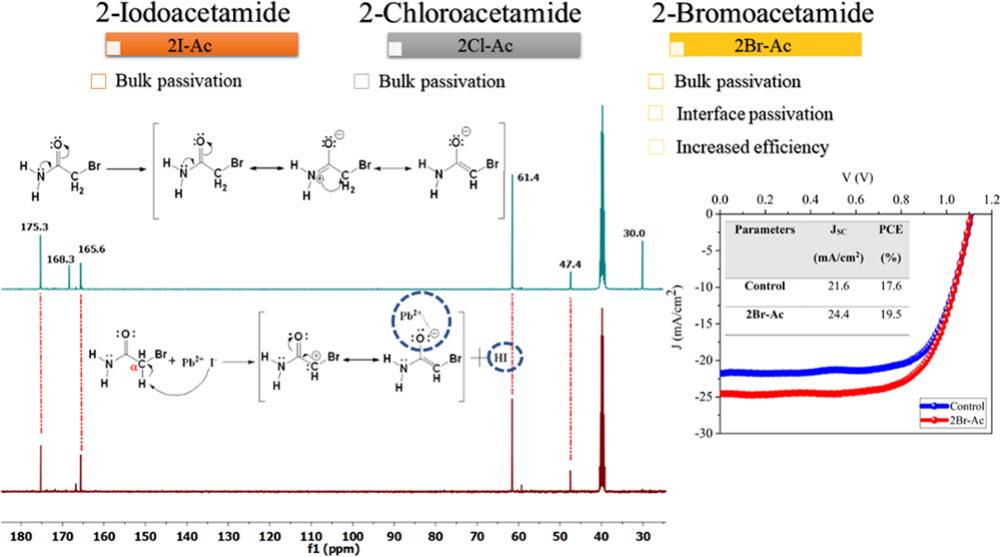

Molecular engineering of additives is a highly effective method to increase the efficiency of perovskite solar cells by reducing trap states and charge carrier barriers in bulk and on the thin film surface. In particular, the elimination of undercoordinated lead species that act as the nonradiative charge recombination center or contain defects that may limit interfacial charge transfer is critical for producing a highly efficient triple-cation perovskite solar cell. Here, 2-iodoacetamide (2I-Ac), 2-bromoacetamide (2Br-Ac), and 2-chloroacetamide (2Cl-Ac) molecules, which can be coordinated with lead, have been used by adding them into a chlorobenzene antisolvent to eliminate the defects encountered in the triple-cation perovskite thin film. The passivation process has been carried out with the coordination between the oxygen anion (−) and the lead (+2) cation on the enolate molecule, which is in the resonance structure of the molecules. The Spiro-OMeTAD/triple-cation perovskite interface has been improved by surface passivation by releasing HX (X = I, Br) as a byproduct because of the separation of alpha hydrogen on the molecule. As a result, a solar cell with a negligible hysteresis operating at 19.5% efficiency has been produced by using the 2Br-Ac molecule, compared to the 17.6% efficiency of the reference cell.

## 1 Introduction

Organic–inorganic hybrid perovskite solar cells (PSCs) have been attracting the most attention among emerging solar technologies due to their low production costs and high-power conversion efficiency. The methylammonium lead iodide (CH_3_NH_3_PbI_3_) based devices, produced by self-assembly on mesoporous titanium dioxide (mp-TiO_2_) films for the first time in 2009, operated with a solar energy conversion efficiency of 3.8%.^1^ The performance development of PSCs has been very rapid due to features such as easily tunable band gaps, high external quantum efficiency, wide absorption spectrum, and long carrier diffusion length.^2−5^ As a result of the studies, a high efficiency value of 25.5% was reached by reducing the charge recombination both in the perovskite layer and in the contact electrodes.^6^ Many methods have been developed to produce high-quality perovskite thin films, such as one- or two-step coating, antisolvent engineering, chemical vapor deposition, and thermal evaporation.^7−13^ Among these methods, antisolvent engineering is a low-cost and easily applicable method for controlling the crystal growth process, increasing the particle size, improving the surface homogeneity, and removing the residual solvents and complexes.^14−18^ However, the performance of such high-performance PSCs is negatively affected by external factors such as humidity, UV, oxygen, and heat and by internal factors such as ion migration defects and removal of volatile organic cations from the surface during the thermal annealing process.^19−22^

One of the effective approaches to eliminate these defects encountered in PSCs is using additives as a passivating agent. An appropriate additive can effectively improve the efficiency and stability of perovskites.^23−25^ Crystal defects, especially anion vacancies and unsaturated lead cations, mostly located at grain boundaries (GBs) and interfaces with charge carrier extraction layers, can cause carrier recombination and ion migration, which reduce device performance and stability.^26,27^ The perovskite crystal, like many other ionic materials, contains poorly coordinated ions at its crystal surfaces as well as at the grain boundaries between individual crystals. In 2019, Zhang *et al.* enhanced the efficiency from 20.22 to 21.02% with the 4-aminobenzonitrile passivation molecule to the triple-cation perovskite precursor solution, hence improving both positive and negative charge defects.^28^ Defects due to leaving halide anion and/or organic cation at grain boundaries in perovskite can act as centers for nonradiative recombination, which is unfavorable for performance and can also cause hysteresis. Uncoordinated Pb atoms due to the missing iodide can also act as electronic trap states^29,30^ that can be passivated by electron-donating Lewis bases such as thiophene or pyridine. In the study by Snaith *et al.*, after passivation by coordination between the sulfur atom in thiophene or the nitrogen atom in pyridine and the poorly coordinated lead ions in perovskite, it decreased the radiative recombination and increased the efficiency of the control cell from 13 to 15.3% for thiophene and to 16.5% for pyridine.^31^ Han *et al.* passivated the defects in the perovskite by adding D-π-A molecules with different electron densities into the CB solvent. It has been shown that PbI_2_ cation defects that are not coordinated with the strong electron-donating *N*,*N*-dibuthylaminophenyl unit in the molecule increase the electron density by binding more tightly, and as a result, the efficiency of the control cell is increased from 18.52 to 20.43%.^32^ The perovskite thin films prepared using the solution method may contain a large number of grain boundaries and defects such as dislocations, impurities, and voids due to the fracture of chemical bonds easily formed at grain boundaries.^26,33^ The pyridine and carboxyl groups on pyridine-2-carboxylic lead salt (PbPyA_2_) not only control crystallization but also passivate the grain boundaries, resulting in a higher-quality perovskite thin film with larger apparent grain boundaries and fewer defects.^34^ Park *et al.* were able to produce high-performance cells with minimal hysteresis by adding the melamine iodide defect passivation molecule into the solution in FA-based PSCs.^35^ In 2018, Dai *et al.* reported the (FAPbI_3_)_0.85_(MAPbBr_3_)_0.15_ device containing the nonvolatile acetamide molecule that exhibited an improved 19.01% efficiency with a higher-quality film, a larger apparent grain size, and less hysteresis compared to the reference cell.^36^ Recently, Yang *et al.* showed that the grain size improved with homogeneous distribution at the grain boundaries using the acetamide molecule in the CH_3_NH_3_PbI_3_ structure.^37^ Han *et al.* passivated the trap states at the mp-TiO_2_/perovskite interface and within the perovskite by adding amide derivatives such as formamide, acetamide, and urea to the CH_3_NH_3_PbI_3_ structure.^38^ To improve the photovoltaic conversion efficiency (PCE) of PSCs, it is necessary to optimize the crystallization processes of perovskite films.^39^ The addition of hydrogen iodide (HI) increases the solubility of PbI_2_ or PbCl_2_ and delays the growth of perovskite crystals, providing high photovoltaic efficiency. Moreover, the HI dopant can provide iodine to fill the halide vacancies and reduce defects on the surface and at the boundary of perovskite crystals, resulting in a reduced recombination between charge carriers.^40,41^

Surface passivation is one of the most studied methods to improve open circuit voltage (*V*_OC_) in solar cells by reducing defects in the bulk or surface of the absorber layer. Suppression of defects formed in the perovskite layer or perovskite/transport layer interfaces has been shown to be critical to further improve the performance of PSCs toward their thermodynamic limits. Numerous studies have been focused on improving the interface between perovskite/electron transport material (ETM) or perovskite/hole transport material (HTM) interface and perovskite layers. Song *et al.*, using dopamine-capped TiO_2_ nanoparticles as ETM via a chelating effect to improve the interfacial bonding with the triple-cation perovskite active layer, significantly reduced the oxygen vacancies and suppressed the deep trap states in TiO_2_. In addition, terminal amino groups in dopamine uncoordinated Pb atoms and reduced Pb-I/Br antisite defects at the triple-cation perovskite/TiO_2_ interface, achieving an efficiency approaching 21%.^42^ Wang *et al.* achieved a 21.46% efficiency, maximizing the passivation effect at the SnO_2_/perovskite interface, which can be predicted by the simultaneous presence of C–N and C=O groups in 3,4-dihydroxyphenylalanine (DOPA).^43^ In the study by Dai *et al.*, methyl ammonium chloride (MACl)-treated film effectively passivated the perovskite interface and hole transport across the film, thus reducing interface recombination. The *V*_OC_ value, which was 1.06 V for the standard cell, increased to 1.09 V when treated with MACl. The fill factor (FF) increased due to the reduction in charge accumulation by the interface dipoles. Therefore, it caused a slight improvement in *J*_SC_ due to efficient electron transport. As a result, PCE increased significantly from 18.78% to a remarkable 20.40% for the MACI-20-treated device.^44^ In another study, Nazeeruddin *et al.* presented a surface passivation layer by spin-coating the formamidinium bromide (FABr) precursor on a film of a mixed perovskite, (FAPbI_3_)_0.85_(MAPbBr_3_)_0.15_, prepared in the presence of excess PbI_2_. Bromide perovskite (FAPbBr_3*x*_I_*x*_) with a wider band gap acts as a barrier for charge carrier recombination at the interface between the mixed perovskite and HTM, resulting in increased *V*_OC_.^45^ You *et al.* used the organic halide salt phenethylammonium iodide (PEAI) on HC(NH_2_)_2_–CH_3_NH_3_ mixed perovskite films for surface defect passivation. They showed that PEAI suppressed nonradiative recombination by reducing defects and could result in more efficient cells as a result.^46^ Byranvand *et al.* modified the perovskite/Spiro-OMeTAD interface by adding a small amount of acetonitrile to the Spiro-OMeTAD precursor solution. As a result, electrical contact is improved, hole collection is increased, and interface recombination losses are reduced.^47^ Hao *et al.* showed that adding an ultrathin tetraphenyldibenzoperiflanthene (DBP) layer to the FA_*x*_MA_1–*x*_PbI_*n*_Br_3–*n*_/Spiro-OMeTAD interface can effectively reduce hole transfer across the FA_*x*_MA_1–*x*_PbI_*n*_Br_3–*n*_/Spiro-OMeTAD interface by smoothing the perovskite surface. It also provides a more favorable hole transport extraction channel due to the horizontal molecular orientation and the matching energy level alignment of the DBP.^48^ All these findings clearly show that the passivation of defects both at interfaces and in bulk is quite important to obtain a high PCE.

In this study, 2-iodoacetamide (2I-Ac), 2-bromoacetamide (2Br-Ac), and 2-chloroacetamide (2Cl-Ac) molecules are exploited to effectively passivate the triple-cation perovskite structure and interface defects simultaneously. Unlike the acetamide molecule used in several studies in the literature,^36^ the most important effect of these molecules is the halogen groups attached to acetamide. While the acetamide molecule provides an increase in efficiency by coordinating on uncoordinated lead, the molecules in this study both coordinate with lead and perform the passivation process by releasing the halogen on it as a result of spontaneous acid–base reactions. The passivation process is carried out by the coordination between the oxygen anion (−) and the lead (+2) cation on the enolate molecule, which is in the resonance structure of the molecules, and the Br atom on the molecule increases the electron cloud density. In addition, HX (X = I, Br) is released as a byproduct as a result of the separation of the alpha hydrogen on the molecule, contributing to the surface passivation process. With a small amount of 2Br-Ac, trap states in both triple-cation perovskite and triple-cation perovskite/Spiro-OMeTAD interface were reduced, charge carrier lifetime was increased, and the short circuit current densities (*J*_SC_’s) of cells produced with triple-cation perovskite were increased. As a result, the highest efficiencies of 19.5% and the negligible hysteresis were attained with 2Br-Ac.

## 2 Results and Discussion

**Figure 1:**
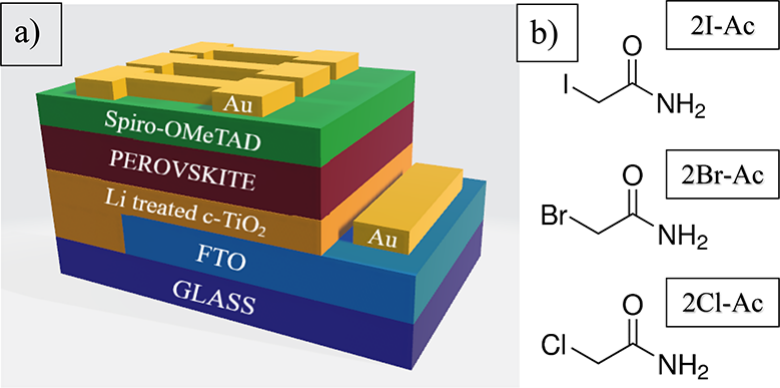
(a) Schematic structure of the triple-cation perovskite solar cell. (b) The structure of 2I-Ac, 2Br-Ac, and 2Cl-Ac molecules.

As seen in Figure 1a, triple-cation PSCs cells with an FTO/Li-treated c-TiO_2_/triple-cation perovskite/Spiro-OMeTAD/Au device architecture was fabricated. The triple-cation perovskite thin films with the composition of Cs_0.05_(FA_0.83_MA_0.17_)_0.95_Pb(I_0.83_Br_0.17_)_3_ were coated with a one-step coating method, and an antisolvent approach was used to control the crystallization dynamics.^49^ An antisolvent washing process was performed with CB for the reference device.^50^ 2I-Ac, 2Br-Ac, and 2Cl-Ac molecules (Figure 1b) were added into CB to perform the passivation process on the triple-cation perovskite thin film. UV–vis absorption, PL, TRPL, XRD, NMR, XPS, cross-sectional SEM, and AFM techniques were used to examine the effects of the three molecules added into CB on the triple-cation perovskite structure.

**Figure 2:**
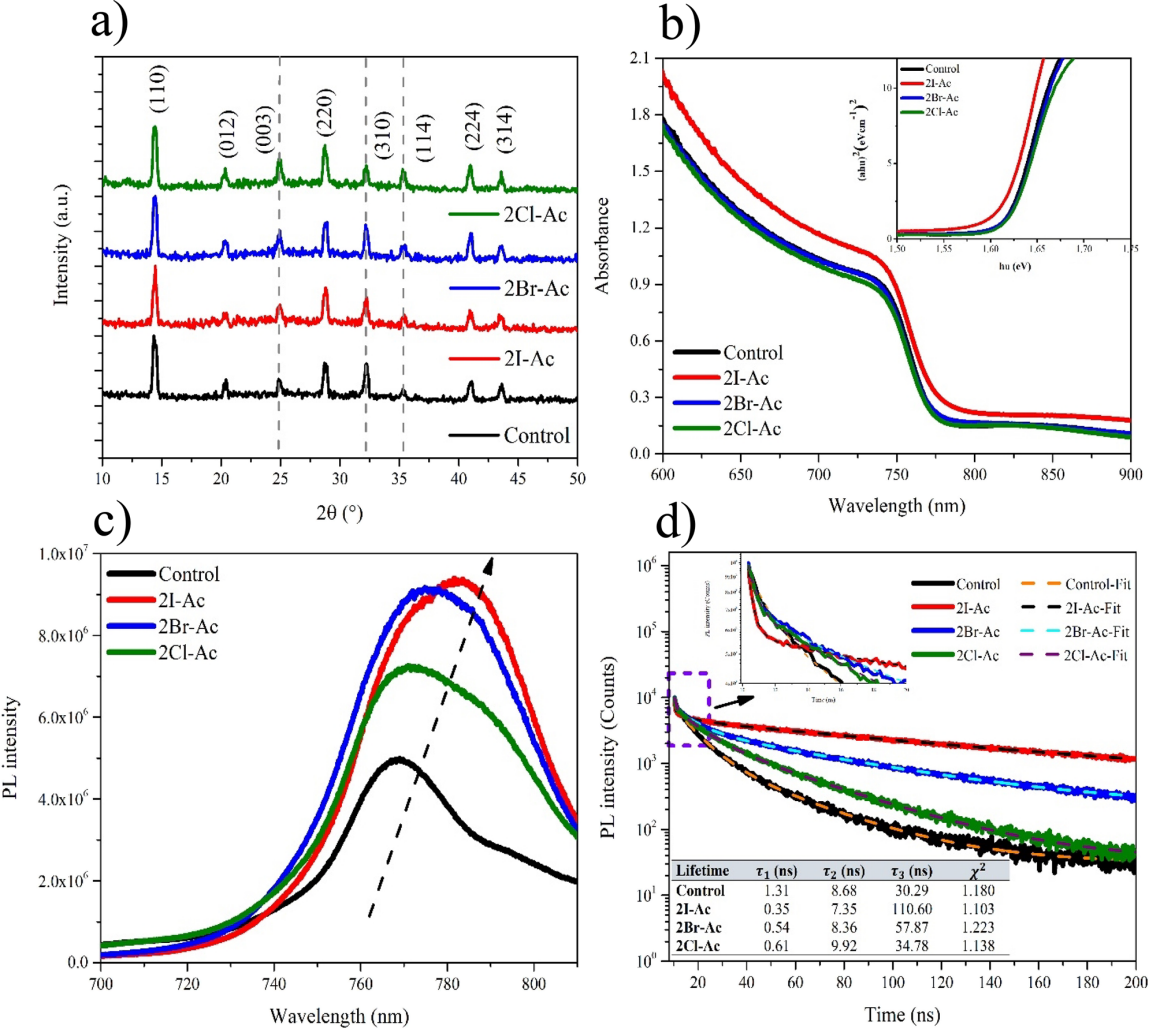
(a) XRD patterns of triple-cation perovskite films without and with three different passivation molecules and (b) UV–vis absorption spectra (Tauc plots are shown in the inset) of triple-cation perovskite thin films without and with 2I-Ac, 2Br-Ac, and 2Cl-Ac molecules. (c) Emission and (d) TRPL spectra of triple-cation perovskite thin films without and with 2I-Ac, 2Br-Ac, and 2Cl-Ac molecules.

X-ray diffraction (XRD) measurements were performed to examine the effects of passivation molecules on the triple-cation perovskite crystal structure. In Figure 2a, the diffraction peaks from all triple-cation perovskite thin films are almost in the same position. Characteristic peaks of 2θ = 14.34, 20.4, 24.84, 28.7, 32.2, 35.3, 40.1, and 43.6° belonging to the triple-cation perovskite structure are observed in CB, 2I-Ac, 2Br-Ac, and 2Cl-Ac washed thin films.^49−51^ It is noteworthy that there is a difference in the peak intensities at 2θ = 24.5, 31.8, and 35.3° corresponding to (003), (310), and (114) planes (Figure S1). There is no change in the characteristic peaks of triple-cation perovskite crystals, indicating that 2I-Ac, 2Br-Ac, and 2Cl-Ac molecules did not incorporate into the triple-cation perovskite lattices, but it is understood that these molecules contribute to the orientation in the (003), (310) and (114) direction of triple-cation perovskite crystals.^52^

In the absorption spectrums in Figure 2b, the absorption edges of all triple-cation perovskite thin films are in almost the same position.^53^ Since the triple-cation perovskite crystal structure is not much affected by the molecules, significant differences in the optical band gap are not expected. The Tauc plots show optical band gaps of 1.616, 1.613, 1.618, and 1.620 eV for CB, 2I-Ac, 2Br-Ac, and 2Cl-Ac, respectively. These very small changes (4 nm for 2I-Ac) in the band gaps confirm the electronic absorption spectra for the Pb halide triple-cation perovskite shifting to a longer wavelength by changing the halide from Cl to Br and I.^54^ The average film thicknesses for triple-cation perovskite films washed with CB, 2I-Ac, 2Br-Ac, and 2Cl-Ac were determined as 530 ± 10, 585 ± 10, 540 ± 5, and 550 ± 50 nm, respectively (Figure S2). Measured thicknesses are the average values of four different samples at four different points. Since the increased triple-cation perovskite thickness leads to a higher light absorption, the absorbance value of the film washed with the 2I-Ac molecule is the highest.^55,56^ The steady-state photoluminescence (PL) spectra obtained from triple-cation perovskite thin films coated on glass using molecules in the CB antisolvent are given in Figure 2c. The PL peak at about 768 nm for the reference triple-cation perovskite structure is consistent with the literature.^49^ It is obvious that thin film PL densities of the triple-cation perovskite thin film coated on glass increased after using 2I-Ac, 2Br-Ac, and 2Cl-Ac molecules, indicating that nonradiative recombination in the triple-cation perovskite layer was highly suppressed.^57−60^ The increased PL density and a spectral shift in triple-cation perovskite thin films prepared with three molecules compared to the control sample can be explained by surface roughness and thin film thickness variations, which favor light coupling and self-absorption, respectively.^61,62^

This spectral shift occurred in the order of 2I-Ac > 2Br-Ac > 2Cl-Ac. Considering the τ_3_ lifetime values, the order 2I-Ac > 2Br-Ac > 2Cl-Ac > control confirms this.^62^ Additionally, Figure 2c shows two emission peaks, where the low-energy emission peak indicates the iodide-rich phase, while the high-energy emission peak corresponds to the remaining mixed halide phase, and the charge carrier lifetime increases due to the trapping of photoexcited carriers in the segregated smaller bandgap iodide-rich domains.^62^ Time-resolved photoluminescence (TRPL) decay measurements were performed with the main PL peak at 768 nm to clarify the effect of these three molecules on the photoluminescence dynamics using the excitation laser with a wavelength of 656 nm, shown in Figure 2d. There are three components occurring in the surface region, the surface–bulk transition region, and the bulk region. These are multiple exponential decay components in which the presence of three decays—τ_1_, τ_2_, and τ_3_—arises. It can be attributed to contributions from τ_1_ excitons, τ_2_ electron–hole pairs, and τ_3_ free charge carriers.^63,64^ The lifetime τ_1_, which is the fastest radiative decay, is quite different for all thin films. Compared to the reference film, the τ_1_ lifetime was considerably reduced after using 2I-Ac, 2Br-Ac, and 2Cl-Ac molecules. It is possible to suppress charge transfer recombination and promote charge transfer by passivation of uncoordinated lead cations, which are mostly located at grain boundaries and at interfaces with charge carrier transport layers.^65^ Since PL emission will increase with the elimination of lead defects, a reduction in τ_1_ is expected. The τ_1_ value of the thin film washed with the 2I-Ac molecule was the lowest, indicating that the surface defects were quite high compared to the others. Since these improved lead defects can no longer trap charge carriers, the probability of radiative recombination in the surface and surface–bulk transition layers will increase. Therefore, a reduction in τ_2_ is expected as well. Also, the diffusion of free carriers into the bulk region is induced, resulting in more fluorescence emission through the free carrier, thereby facilitating carrier transport and prolonging the free-carrier lifetime (τ_3_).^63^ It was found that the triple-cation perovskite films washed with 2I-Ac, 2Br-Ac, and 2Cl-Ac molecules exhibited a longer τ_3_ than that of the control sample as shown in Figure 2d. The significantly extended lifetime of the triple-cation perovskite films with these three molecules represents the successful suppression of defects by the severe carrier nonradiative recombination in the triple-cation perovskite bulk. As shown in Figure 2d, the TRPL measurement shows that the τ_3_ lifetime is increased from 30.29 to 110.60, 57.87, and 34.78 ns in washing with 2I-Ac, 2Br-Ac, and 2Cl-Ac molecules, respectively. Reducing nonradiative recombination would allow devices to reach higher open circuit voltages and PCE. These PL and TRPL results show significant suppression of carrier recombination and traps with 2Br-Ac, which benefits the charge transport process. PL and TRPL measurements show that passivation with 2Br-Ac successfully passivated the defect-assisted recombination in the triple-cation perovskite active layer.^66^ The triple-cation perovskite film on the glass substrate has the highest PL density, indicating significant carrier recombination in the film. Clearly, the photo-quenching efficiency of 2Br-Ac/Spiro-OMeTAD is greater than the others and shows the most effective charge extraction (Figure S3). Although a higher τ_3_ lifetime was obtained from the film produced with the 2I-Ac molecule, it negatively affected the charge transfer at the triple-cation perovskite/Spiro-OMeTAD interface, as it had more surface defects.^67^ The insufficient charge transfer at the triple-cation perovskite/Spiro-OMeTAD interface formed with the 2I-Ac molecule was supported by both PL and EIS measurements.

Nuclear magnetic resonance (NMR) experiments were performed using 2Br-Ac with PbI_2_ to shed light on the interaction of the molecules with the triple-cation perovskite structure. 2Br-Ac is an amide derivative molecule containing a bromine atom at the alpha carbon hydrogen atom attached to the alpha carbon named alpha hydrogen that shows acidic properties due to the molecule’s resonance stability. Hydrogen atoms attached to the nitrogen atom in the amide functional group can form hydrogen bonds with polar aprotic solvents such as DMSO. Under these conditions, the molecule forms a resonance structure called an enolate.^68,69^ It is known in the literature that the enolate molecule can coordinate with metal atoms. In this experiment, the resonance structures formed by the 2Br-Ac molecule in the presence of DMSO-*d*_6_ and the coordination compound formed in the presence of PbI_2_ were examined using ^13^C NMR spectroscopy.^68^ In the 2Br-Ac ^13^C NMR spectrum obtained only with DMSO-*d*_6_ solvent, a chemical shift of carbonyl carbon (−C=O), methylene carbon (−CH_2_), amine carbon (−C=N), and carbonyl carbon in the enolate structure and, furthermore, an alkene double bond and a neighbor carbonyl functional group were observed at 165.6, 47.4, 168.3, 30.0, 175.3, and 61.4 ppm, respectively. When 10 mg of PbI_2_ was added to the medium, it was observed that the chemical shifts of (−C=N) and neighbor carbanion carbon atoms at 168.3 and 30.0 ppm were suppressed.

**Figure 3:**
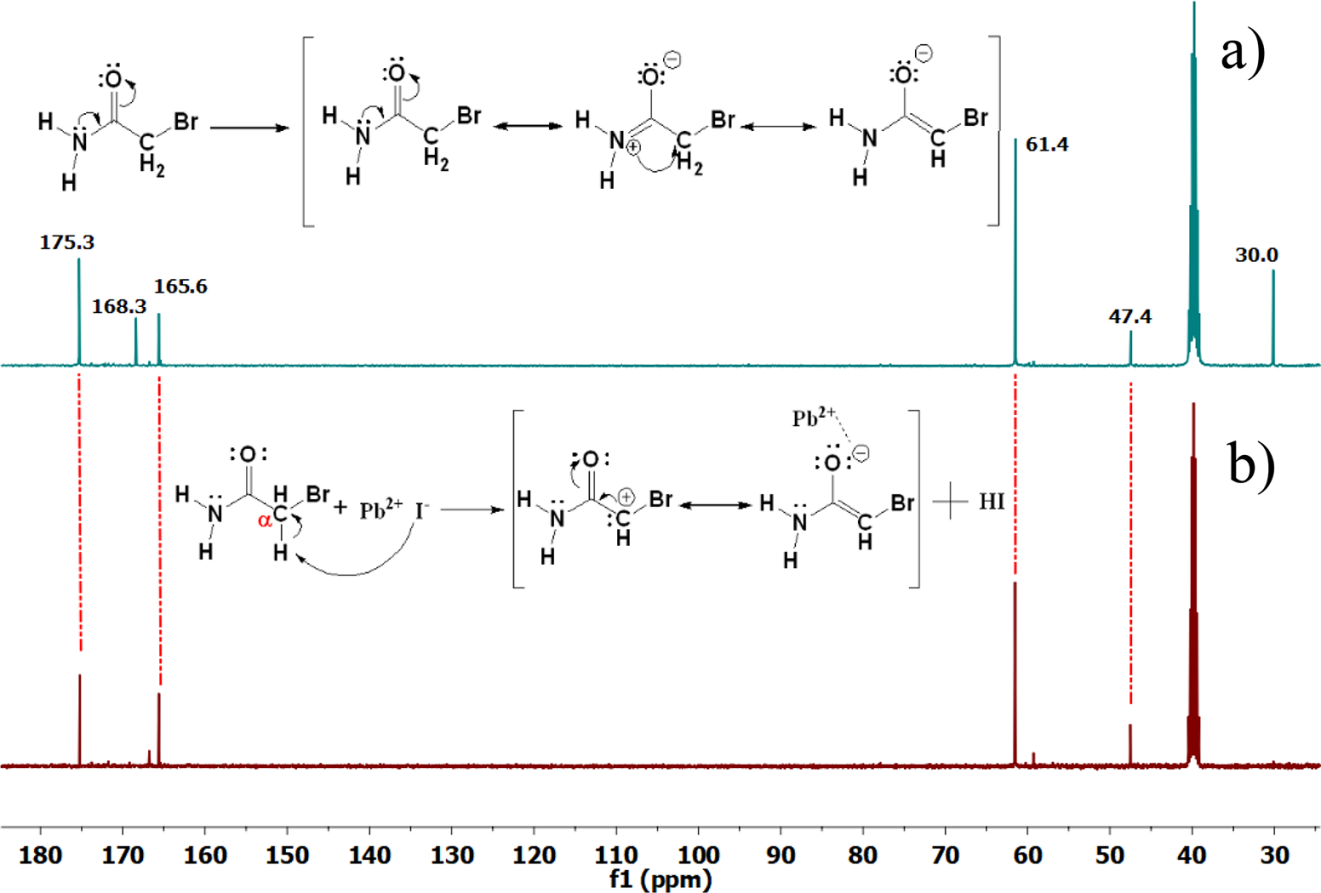
^13^C NMR spectra of the (a) 2Br-Ac and (b) 2Br-Ac + PbI_2_ solution in DMSO-*d*_6_.

This situation can be explained by the reaction between the iodide and alpha hydrogen on the 2Br-Ac molecule. The alpha hydrogen on carbonyl compounds shows acidic properties due to its resonance stability on the molecule. I^–^ in the medium shows a basic character. Therefore, an acid–base reaction takes place between them.^70^ As a result of the reaction, chemical shifts of two resonance structures were observed unlike the ^13^C NMR spectrum obtained only with the DMSO-*d*_6_ solvent. The NMR results confirmed the interaction between the triple-cation perovskite and 2Br-Ac molecule. As a result of the reaction, while new resonance structures are formed in the medium, Pb^+2^ cations stabilize the enolate structure through coordination with oxygen and cause it to be dominant in the medium (Figure 3).^68^ Thus, in the ^13^C NMR spectrum, the signals at 175.3 and 61.49 ppm appear, while other signals remained unchanged at the same chemical shift values. To better understand the lead complex between 2Br-Ac and PbI_2_, ^1^H NMR spectra were recorded using the 2Br-Ac and 2Br-Ac + PbI_2_ solution in the DMSO-*d*_6_ solvent. Because of the intramolecular interaction of amine protons, protons at the aromatic region of the spectra were observed as two singlets for both samples (Figures S4 and S5).^71^ Most importantly, no signal shifts were observed. ^13^C NMR results indicate that the Pb^+2^ cations in the perovskite layer readily bond with the partially negative oxygen atoms.^72^ According to this result, the carbonyl oxygen on the enolate resonance structure instead of the amine group is favored by the Pb^+2^ cation in the solution phase.

**Figure 4:**
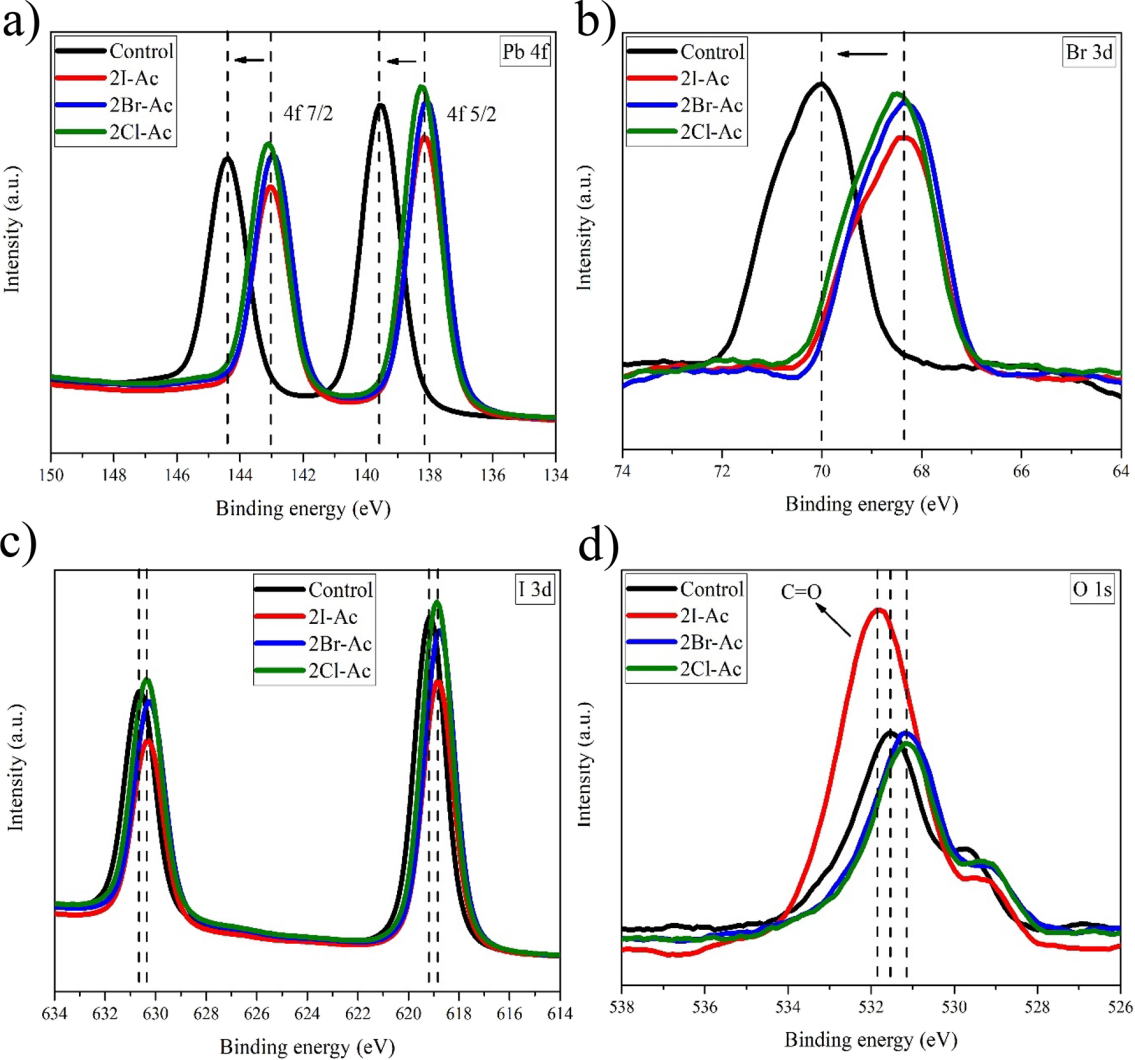
High-resolution XPS spectra of (a) Pb 4f, (b) Br 3d, (c) I 3d, (d) and O 1s peaks of triple-cation perovskite thin films without and with 2I-Ac, 2Br-Ac, and 2Cl-Ac molecules.

The X-ray photoelectron spectroscopy (XPS) technique was used to obtain more evidence about the interaction of molecules with elements in the triple-cation perovskite structure. The XPS full survey spectra of the FTO/triple-cation perovskite films washed with CB, 2I-Ac, 2Br-Ac, and 2Cl-Ac can be found in Figure S6. High-resolution XPS peaks of Pb 4f, I 3d, Br 3d, O 1s, and N 1s spectra are given in Figure 4 and Figure S8. The Pb 4f spectrum for the control sample in the triple cation perovskite structure shows two distinct peaks, such as 4f 5/2 (144.38 eV) and 4f 7/2 (139.58 eV), and these binding energies are consistent with the literature.^73,74^ Upon the addition of 2I-Ac, 2Br-Ac, and 2Cl-Ac molecules into the triple-cation perovskite thin films, the Pb 4f peaks shift to binding energies 1.5 eV lower than the control sample, such as 4f 5/2 (142.98 eV) and 4f 7/2 (138.18 eV), indicating the interaction between Pb^+2^ ions and molecules on the triple-cation perovskite surface, which is in line with the NMR results (Figure 4a). According to the results obtained from ^13^C and ^1^H measurements, the Pb^+2^ ion is in coordination with oxygen. Thus, the Pb–O coordination will significantly affect the charge transfer between the triple-cation perovskite and the molecules.^75^ The peaks of the Br 3d signal are located at two peaks with binding energies varying between 64.28 and 68.53 eV (3d5/2) and 68.61 and 69.39 eV (3d3/2) corresponding to the inner and surface bromine ions, respectively, as shown in Figure 4b.

In addition, the fitted peaks of the Br 3d signal are summarized in Table S1 and Figure S7a–d. Compared with the control triple-cation perovskite, the Br 3d signals shifted to lower binding energies of 1.70 eV for the passivated triple-cation perovskite with 2I-Ac, 2Br-Ac, and 2Cl-Ac molecules. The intensity ratio of the Br 3d3/2 peak to the 3d5/2 peak is drastically higher for the 2Br-Ac passivated triple-cation perovskite film, indicating a Br-rich surface.^76^ In Figure 4c, I 3d has two binding energies: 3d5/2 (619.18 eV) and 3d 3/2 (630.68 eV). These two peaks shifted to smaller binding energies of about 0.54 eV when passivated by the three molecules. In Figure 4d, the C=O position is located at 531.55 eV in the spectrum of O 1s taken from the reference triple-cation perovskite surface.^77^ When passivation was performed with 2Br-Ac and 2Cl-Ac molecules, there was a shift of 0.45 eV to the smaller binding energy. In the 2I-Ac molecule, there was a shift to the higher binding energy of about 0.25 eV. By passivating the triple-cation perovskite thin film with the 2I-Ac molecule, the binding energy of the electron acceptor C=O carbonyl group shifted to a larger binding energy. Since I^–^ has the lowest electronegativity compared to Br^–^ and Cl^–^, it shows that the charge transfer between the triple-cation perovskite and 2I-Ac is slower from the 2Br-Ac and 2Cl-Ac. According to the device performances, this suggests that the electron cloud density distribution on 2I-Ac negatively affects the charge transfer between the molecule and the triple-cation perovskite; as a result, the lowest *J*_SC_ value was obtained.^38^ The N 1s core-level binding energy is observed at 400.7 eV and assigned to the N atoms in the FA cation in the mixed cation perovskite film.^78^ The N 1s peaks in the perovskite structure modified with the three molecules shifted to lower binding energies compared to the control sample (Figure S8). The shift in N 1s binding energies is the same for 2I-Ac and 2Cl-Ac molecules (400.2 eV) in the modified thin film, while it is greater for 2Br-Ac (400.1 eV).

**Figure 5:**
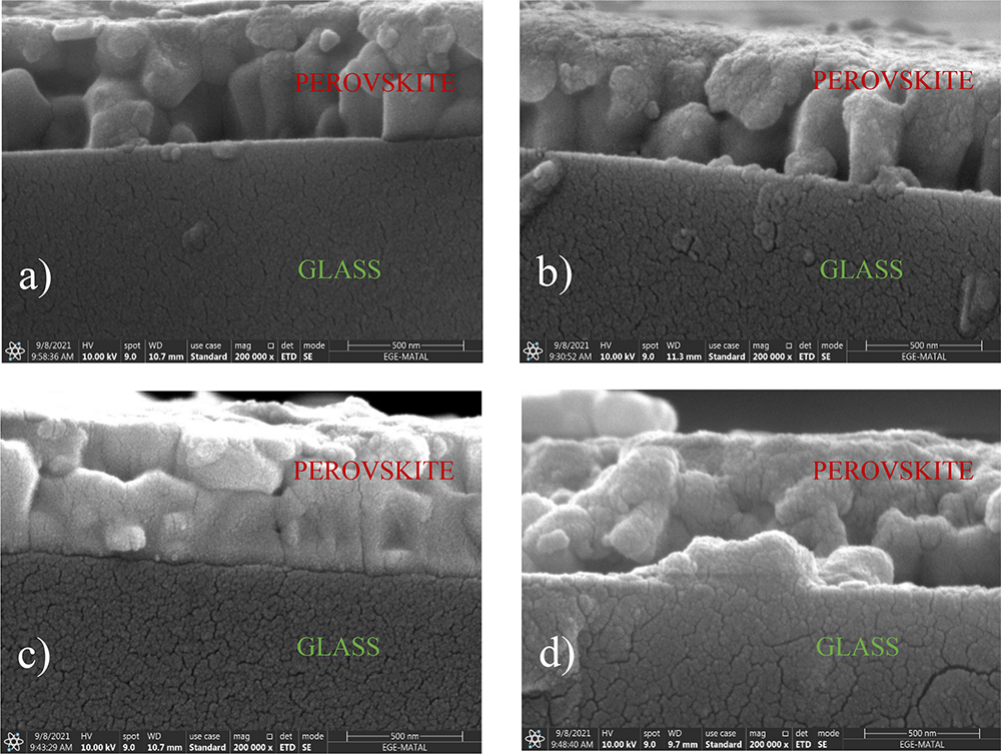
SEM cross-sectional images of triple-cation perovskite thin films prepared with (a) CB, (b) 2I-Ac, (c) 2Br-Ac, and (d) 2Cl-Ac.

Morphological properties of triple-cation perovskite thin films were investigated using atomic force microscopy (AFM) and scanning electron microscopy (SEM). Figure S9 shows the AFM topographic and 3D images of the triple-cation perovskite thin film before and after passivation. The root mean square (RMS) value of the reference film washed with CB was 10.6 nm, while it was 16.2, 12.1, and 11.6 nm for 2I-Ac, 2Br-Ac, and 2Cl-Ac, respectively. All triple-cation perovskite film surfaces have a very homogeneous and smooth morphology. The apparent grain size of the triple-cation perovskite thin film washed with 2I-Ac is quite large compared to the reference film.^61^ To explore the passivation effect of 2I-Ac, 2Br-Ac, and 2Cl-Ac molecules on the triple-cation perovskite layer, we examined the SEM cross-sectional images of the thin films. As shown in Figure 5, we observed that triple-cation perovskite grains are more homogeneous, smooth, and large for the devices prepared with 2Br-Ac.

From the cross-sectional SEM images of triple-cation perovskite thin films, we find that the triple-cation perovskite grains are tightly stacked and become larger with the passivation of 2Br-Ac compared to others. At the middle of the triple-cation perovskite layer prepared with control, 2I-Ac, and 2Cl-Ac, the horizontal triple-cation perovskite grain boundaries are more apparent and smaller, while there are fewer horizontal grain boundaries for the 2Br-Ac passivated triple-cation perovskite absorber. The structure with less horizontal apparent grain boundaries would be suitable for carrier diffusion and superior photovoltaic performance.^79^ This can support the high FF and *J*_SC_ of devices manufactured with 2Br-Ac, as more uniform grains enable better charge transfer. There are fewer voids or defects throughout the triple-cation perovskite prepared with 2Br-Ac compared to the other films.

**Figure 6:**
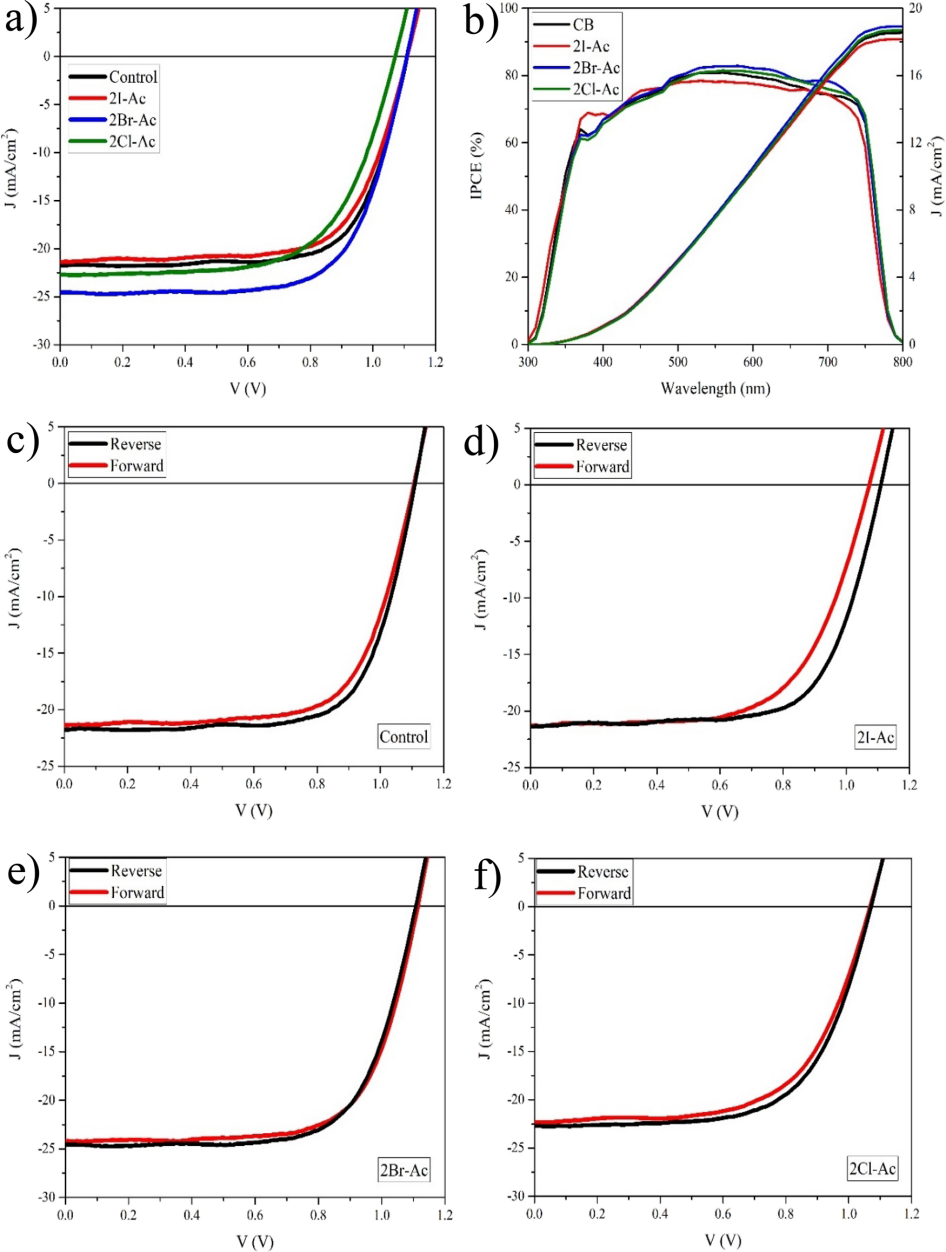
(a) Current density vs voltage (*J*–*V*) of the best solar cells without and with 2I-Ac, 2Br-Ac, and 2Cl-Ac. (b) IPCE spectra of the best solar cells without and with 2I-Ac, 2Br-Ac, and 2Cl-Ac. *J*–*V* plots of triple-cation perovskite devices obtained by (c) control, (d) 2I-Ac, (e) 2Br-Ac, and (f) 2Cl-Ac under forward and reverse bias.

To examine the effects of 2I-Ac, 2Br-Ac, and 2Cl-Ac molecules, the PSCs in the n-i-p structure were fabricated as in Figure 1. The best photovoltaic parameters values obtained under reverse and forward bias are given in Figure 6 and Table 1. The highest photovoltaic conversion efficiency was obtained from the solar cell fabricated with 2Br-Ac under forward and reverse bias. For 2Br-Ac, the short circuit current density (*J*_SC_) increased from 21.6 to 24.4 mA/cm^2^. The addition of 2Br-Ac increased the PCE from 17.6 to 19.5%, respectively, compared to the control device.

**Table 1:** Forward and Reverse Photovoltaic Performance of the PCS Devices Washed with CB, 2I-Ac, 2Br-Ac, and 2Cl-Ac (Hysteresis (HI) Calculated According to )

	scan direction	*J*_SC_ (mA/cm^2^)	*V*_OC_ (mV)	FF (%)	best PCE (%)	HI
control	forward	21.3	1110	69.4	16.4	
	reverse	21.6	1112	73.3	17.6	0.022
2I-Ac	forward	21.3	1075	64.2	14.7	
	reverse	21.3	1112	69.7	16.5	0.036
2Br-Ac	forward	24.3	1112	70.4	19.0	
	reverse	24.4	1112	71.9	19.5	0.008
2Cl-Ac	forward	22.4	1075	61.2	15.2	
	reverse	22.6	1075	66.3	16.1	0.013

**Figure 7:**
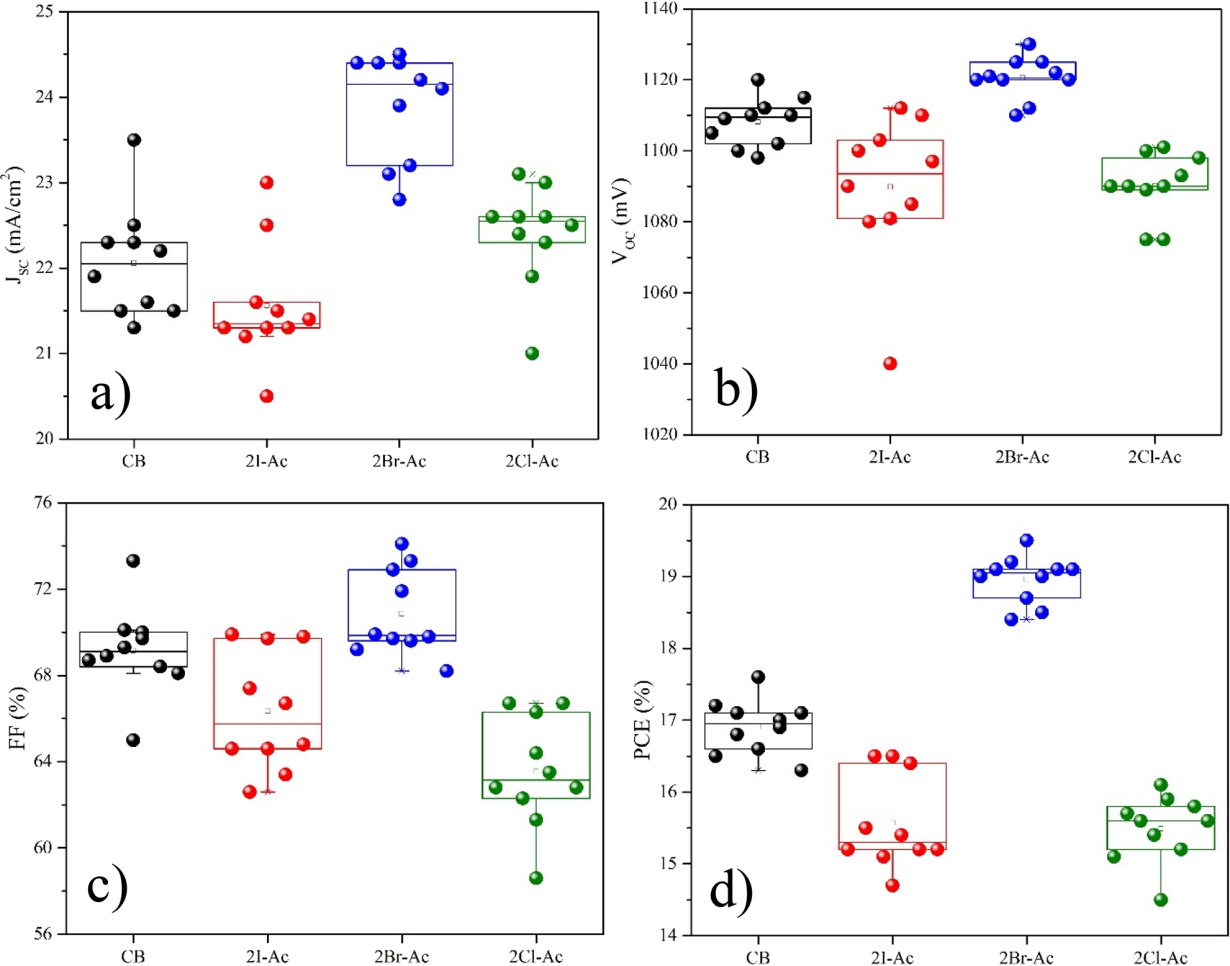
Device performance statistics of the planar solar cells. (a) *J*_SC_, (b) *V*_OC_, (c) FF, and (d) PCE of the triple-cation perovskite solar cells prepared with CB, 2I-Ac, 2Br-Ac, and 2Cl-Ac. Device parameters are obtained from reverse bias.

The bromide-rich triple-cation perovskite surface prepared with the 2Br-Ac molecule resulted in the improvement of *V*_OC_, *J*_SC_, and FF by reducing the charge carrier recombination at the triple-cation perovskite/Spiro-OMeTAD interface. The integrated short-current density was evaluated from the incident-photon-to-current efficiency (IPCE) spectra to be 19.2, 19.0, 18.8, and 18.4 mA/cm^2^ for 2Br-Ac, 2Cl-Ac, control, and 2I-Ac, respectively, which are in good agreement with the *J*_SC_ obtained from the *J*–*V* characteristics (Figure 6b). The deviations between the integral *J*_SC_ values calculated from the IPCE curves and those obtained from the *J*–*V* tests are in the range of 12–21%, and these values are comparable to the errors in previous reports (Table S2).^80^ Theoretically, these deviations can mainly be attributed to the following three reasons: (1) There is an approximately 10–15% loss in IPCE due to the reflection and absorption of FTO glass. (2) Full irradiation applied in the *J*–*V* test generates excess charge carriers and provides more efficient charge transport/collection than wavelength-dependent light in IPCE measurement. (3) Full irradiation includes near-infrared light and thus generates heat, and an increase in temperature leads to an increase in *J*_SC_, while wavelength-dependent light in the IPCE measurement exerts only such an effect.^80−82^ The *V*_OC_ value in the cell fabricated with 2Cl-Ac is lower than the other cells under both forward and reverse bias. The device fabricated with 2Br-Ac has a negligible hysteresis compared to the others (Figure 6c–f and Table 1). Additionally, to verify the reproducibility of the devices, device performance statistics and average PCEs of triple-cation perovskite solar cells prepared with CB, 2I-Ac, 2Br-Ac, and 2Cl-Ac are given in Figure 7 and Table S2. The low *V*_OC_ value of these devices prepared with 2Cl-Ac can be attributed to several reasons. First, according to EIS results, the trap densities at Spiro-OMeTAD/triple-cation perovskite interface are the highest. Second, the solubility of the 2Cl-Ac molecule in CB is relatively low compared to the other molecules. Although total solubility in CB is achieved under 50 °C using the ultrasonic bath, capillary growth occurs due to solution molecule interaction when it cools down to room conditions (Figure S10a). Visible defects occur on the surface of the devices produced using the 2Cl-Ac molecule (Figure S10b).

**Figure 8:**
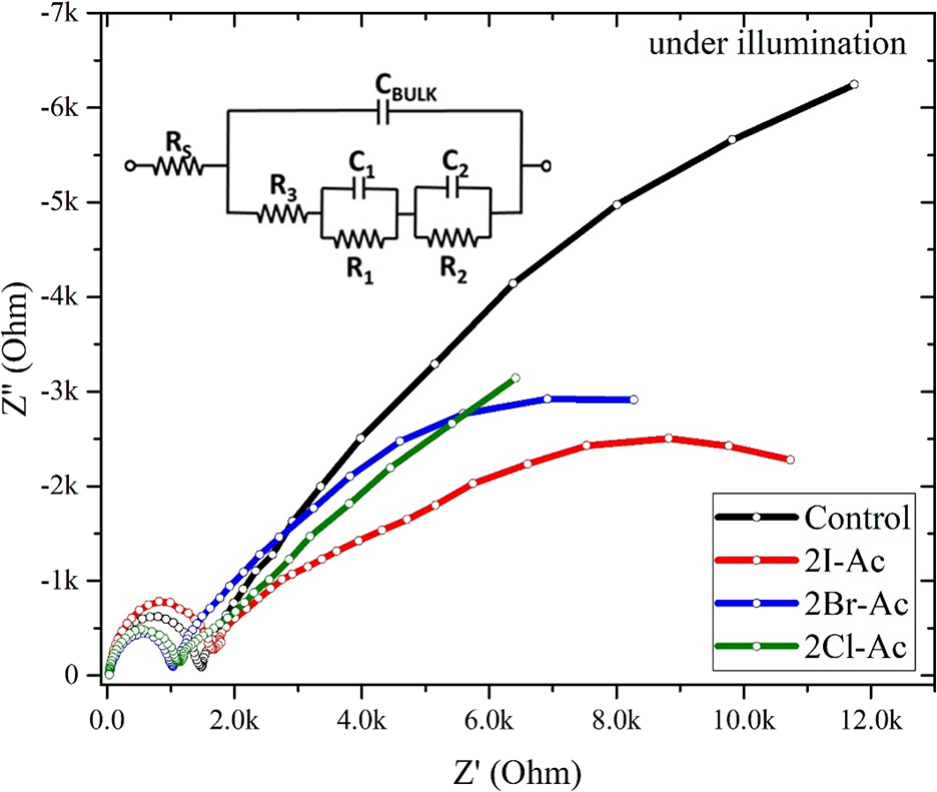
The Nyquist plot of devices under illumination and equivalent circuit to interpret the impedance data.

Impedance spectroscopy (IS) is a powerful and widely used tool for characterizing charge dynamics in perovskite solar cells. An equivalent circuit consisting of resistive and capacitive elements compatible with the physical mechanism of the device is used to interpret impedance spectroscopy data correctly. In 2016, Bisquert *et al.* investigated the correlation of circuit elements with physical processes using different models in the perovskite solar cell.^83^ They reported that the accumulation behavior at the TiO_2_/perovskite and Spiro-OMeTAD/triple-cation perovskite interface could be explained by the equivalent circuit model in Figure 8. According to the model, the point that intersects the real axis in the high-frequency (HF) region in the Nyquist spectrum gives the series resistance (*R*_s_). The arc in the high-frequency region is related to the bulk capacitance (*C*_BULK_) coupled with resistance *R*_3_. *C*_BULK_ is due to the internal dielectric relaxation of the perovskite bulk layer, and *R*_3_ is related to the conductivity of the bulk perovskite. Capacitance and resistance couples *C*_1_*R*_1_ and *C*_2_*R*_2_ are responsible for the large semicircle in the intermediate-frequency (IF) and low-frequency (LF) regions, respectively. They give information about charge accumulation and transfer at the external interface of the perovskite layer.^84,85^ The *R*_1_*C*_1_ couple is associated with the TiO_2_/perovskite interface, while the *R*_2_*C*_2_ couple is relevant with the Spiro-OMeTAD/perovskite interface. The electron diffusion length correlates with Ld2 ∼ τ_rec_/τ_tr_. For charges to reach respective electrodes efficiently, the recombination lifetime (τ_rec_) value is expected to be high and the transport lifetime (τ_tr_) value is expected to be low. However, surface recombination at the outer interface of perovskite causes a decrease in τ_rec_, while nonradiative recombination in bulk affects τ_tr_.

τ_tr_ is obtained by the product of *R*_3_ and *C*_BULK_ in the high-frequency region. Defects in the crystal structure play a significant role in *J*_SC_, leading to a loss of excess carriers and hence increasing τ_tr_.^85^ When defects are located at grain boundaries, the electrical resistivity of the bulk increases. According to the equivalent circuit, *R*_3_ is directly related to bulk resistivity and hence the defects at grain boundaries. The values of *R*_3_ and τ_tr_ are summarized in Table 2. τ_tr_ of the control cell was 2.26 × 10^–5^ s, whereas 2Br-Ac, 2Cl-Ac, and 2I-Ac additives decreased the nonradiative recombination rates in bulk, enhancing to 1.26 × 10^–5^, 1.82 × 10^–5^, and 2.05 × 10^–5^ s, respectively. The 2Br-Ac additive gave rise to an efficient transport of the photocurrent, leading to the highest *J*_SC_ value.

**Table 2:** Extracted Fitting Parameters from Impedance Measurements

fitting parameters	control	2I-Ac	2Br-Ac	2Cl-Ac
*R*_s_ (Ω)	34.60	39.00	33.90	35.00
*C*_BULK_ (10^–8^ F)	1.54	1.26	1.31	1.74
*R*_3_ (Ω)	1379.35	1625.30	958.07	1044.40
*C*_1_(10^–5^ F)	102.97	0.84	5.45	2.98
*R*_1_ (Ω)	393.135	1900	4413.451	373.6809
*C*_2_ (10^–5^ F)	6.02	6.28	11.90	15.80
*R*_2_ (Ω)	21,719.28	9449	5429.029	16,880.1
τ_tr_ (10^–5^ s)	2.26	2.05	1.26	1.82
τ_rec_ (s)	0.89	0.21	0.89	0.32

Another dominant recombination process is surface recombination at the perovskite interface, which directly affects the *V*_OC_. The formation of accumulation zone behaves like a space charge region, and nonradiative carrier recombination at the interface limits the *V*_OC_. The lowest recombination rate in the middle-frequency region at the TiO_2_/perovskite interface (*C*_1_*R*_1_ pair) was obtained with the reference cell. The 2Br-Ac, 2I-Ac, and 2Cl-Ac additives in the washing solvent caused the increase in the density of majority carriers on the outer surface of the perovskite. Therefore, the surface recombination rate increased. However, when the low-frequency region, which gave information about the Spiro-OMeTAD/perovskite interface (*C*_2_*R*_2_ pair), was examined, the recombination rate decreased significantly with 2Br-Ac passivation. The recombination lifetimes, calculated from the middle- and low-frequency regions, for the reference cell, 2Br-Ac, 2Cl-Ac, and 2I-Ac passivated devices were 0.89, 0.89, 0.32, and 0.21 s, respectively. Thus, the trend of *V*_OC_, depending on the surface nonradiative recombination rate, was ranked as *V*_OC_-2Br-Ac = *V*_OC_-CB > *V*_OC_-2Cl-Ac > *V*_OC_ -2I-Ac. According to all these results, reducing the recombination rate at the Spiro-OMeTAD/ triple-cation perovskite interface and improving the charge transport in bulk with the 2Br-Ac addition resulted in the highest device performance with high *J*_SC_ and *V*_OC_.

## 3 Conclusions

In this study, the passivation effects of 2I-Ac, 2Br-A,c and 2Cl-Ac molecules on the triple-cation perovskite structure were investigated. It has been shown that charge traps and defective regions in the triple-cation perovskite layer that act as the nonradiative recombination center are effectively reduced by the treatment of the triple-cation perovskite thin film with the 2Br-Ac molecule without affecting its morphology and crystal structure. Defects in the triple-cation perovskite structure were effectively passivated with 2I-Ac, 2Br-Ac and 2Cl-Ac molecules. The nonradiative charge carrier recombination was reduced, as evidenced by the increase in densities of PL. From TRPL measurements, it was found that triple-cation perovskite films washed with 2I-Ac, 2Br-Ac, and 2Cl-Ac molecules exhibited a longer free carrier lifetime (τ_3_) than the control sample. Although it was shown that nonradiative recombination was successfully suppressed in the triple-cation perovskite active layer passivated with 2I-Ac, 2Br-Ac, and 2Cl-Ac according to PL and TRPL measurements, the grains in the SEM cross-sectional image of the triple-cation perovskite thin film prepared with 2Br-Ac were found to be more homogeneous, smooth, tightly packed, and larger than the others. There are less horizontal grain boundary and fewer voids or defects throughout the triple-cation perovskite prepared with 2Br-Ac. Horizontal grain boundaries in the middle of the triple-cation perovskite layer prepared with CB, 2I-Ac, and 2Cl-Ac are more distinct and smaller. In addition, it was explained by XPS analysis that the triple-cation perovskite thin film surface was passivated with HX (X = I, Br), which occurs as a byproduct of the reaction. The intensity ratio of the Br 3d3/2 peak to the 3d5/2 peak in the Br 3d spectrum is higher than the nonpassivated surface. The increasing surface/inner ratio of Br 3d in the triple-cation perovskite film prepared with 2Br-Ac indicates that 2Br-Ac passivation promotes Br vacancy reduction via blocking the loss of surface Br^–^. As a result of Pb–O coordination, by improving the Spiro-OMeTAD/triple-cation perovskite interface, charge collection was enhanced and recombination at the interface was reduced. In the EIS analysis, a significant reduction of the recombination rate with 2Br-Ac passivation in the low-frequency region corresponding to the Spiro-OMeTAD/triple-cation perovskite interface also confirms this result. The reduced recombination rate and improved charge transfer at the Spiro-OMeTAD/triple-cation perovskite interface with 2Br-Ac passivation explain the high *J*_SC_ and *V*_OC_. Devices manufactured with 2Br-Ac have less horizontal grain boundaries, resulting in carrier diffusion and superior photovoltaic performance, explaining their high FF and *J*_SC_ because more uniform particles provide better charge transfer. As a result, a maximum efficiency of 19.5% was obtained with negligible hysteresis from the cell passivated with 2Br-Ac.

## 4 Experimental Section

### 4.1 Materials

The 2.5 × 2.5 cm^2^ fluorine-doped tin oxide glass slides (FTO OPV-FTO22-15, sheet resistance of 14 Ω·sq^–1^) were purchased from OPVTech. Titanium isopropoxide (Ti[OCH(CH_3_)_2_]_4_, 97%), hydrochloric acid (HCl, 37%), 2-propanol (for HPLC, 99.9%), chlorobenzene (CB, anhydrous 99.8%), acetonitrile (ACN, 99.8%), 4-*tert*-butylpyridine (TBP, 98.0%), and cesium iodide (CsI, 99.999%) were purchased from Sigma Aldrich. Lead iodide (PbI_2_, 99.99%) was purchased from Tokyo Chemical Industry (TCI). PbBr_2_ (99.999%), methyl ammonium bromide (MABr, >99.5%), and formamidinium iodide (FAI, >99.5%) were purchased from Lumtec. Bis(trifluoromethane)sulfonimide lithium salt (Li-TFSI, 99.0%), *N*,*N*-dimethylformamide (DMF, anhydrous >99.5%), 2-bromoacetamide (98%), and 2-chloroacetamide (98%) were purchased from Acros. Dimethly sulfoxide (DMSO, >99.7%), 2-iodoacetamide (98%), and dimethyl sulfoxide-*d*_6_ (DMSO-*d*_6_) were purchased from Merck. Spiro-OMeTAD was purchased from Borun New Material Technology. All chemical substances obtained commercially were used as purchased without further purification.

### 4.2 Device Fabrication

The 2.5 × 2.5 cm^2^ conductive fluorine-doped tin oxide (FTO) substrates were subjected to a 20 min cleaning procedure with distilled water, acetone, and alcohol, respectively. A compact titanium dioxide (TiO_2_) layer used as an electron carrier layer was prepared by the sol–gel method. To remove organic contamination remaining on the surface of the cleaned conductive FTO glasses, all films were exposed to oxygen plasma for 7 min. Then, all films were annealed on a hot plate at 460 °C for 1 h. The Li-TFSI solution (0.1 M) prepared in acetonitrile was coated on TiO_2_-coated films for 40 s at 3000 rpm and annealed on the hot plate at 460 °C for 1 h.^86^ To form the triple-cation perovskite precursor solution, PbI_2_ (1.1 M), PbBr_2_ (0.22 M), MABr (0.2 M), FAI (1 M), and CsI (0.05 M) materials were prepared by mixing in a DMSO/DMF (1:4) solvent system. The triple-cation perovskite solution was coated on Li-treated TiO_2_ films for 25 s at 2500 rpm and 20 s at 5000 rpm. Anhydrous CB (100 μL) was carefully added dropwise to the surface of the triple-cation perovskite during the spin coating at the second step for 5 s, and the substrates were annealed at 100 °C for 1 h on the hot plate in the glovebox. For passivation of 2I-Ac, 2Br-Ac, and 2Cl-Ac, each was added separately at 1 mg/1 mL into CB. To increase the solubility of the molecules in chlorobenzene, the solutions were kept in an ultrasonic bath for 20 min at 50 °C. There is a solubility problem at concentrations higher than 1 mg/1 mL. Triple-cation perovskite thin films washed separately with CB and CB + molecules were annealed on the hot plate for 1 h at 100 °C. Spiro-OMeTAD (in 73 mg/1 mL anhydrous CB) was coated at 4000 rpm for 20 s after adding 16 μL of the Li-TFSI/anhydrous acetonitrile solution (520 mg/mL) and 30 μL of tBP. Finally, gold (80 nm) was deposited on top of Spiro-OMeTAD via thermal evaporation under a high vacuum at 3 × 10^–7^ Torr. The cell active area was fixed at 0.095 cm^2^.

### 4.3 Characterization

The current density–voltage (*J*–*V*) curves were recorded under ambient temperature by a solar simulator with a digital Keithley 2400 source meter without any device preconditioning. An AM1.5G filter was used as the light source, and a light power of 100 mW/cm^2^ was used in all *J*–*V* measurements. Before each measurement, the exact light intensity was determined with a calibrated silicon reference solar cell (area 4 cm^2^). The average photovoltaic parameters of the cells were calculated from 10 devices. The incident-photon-to-current efficiency (IPCE) measurements were recorded with the Enlitech QE-R system. The surface morphological properties of annealed triple-cation perovskite films (control and treated with 2I-Ac, 2Br-Ac, and 2Cl-Ac molecules) were determined with atomic force microscopy (AFM, Park Systems NX20, PPP-NCHR 5M) in noncontact mode. The cross-sectional images of triple-cation perovskite thin films were characterized using a scanning electron microscope (SEM, Thermo Scientific Apreo S). The crystallinity and purity of the annealed triple-cation perovskite films (control and treated with 2I-Ac, 2Br-Ac, and 2Cl-Ac molecules) were collected by a Rigaku XRD Ultima IV diffractometer. An IM6 electrochemical workstation (Zahner Co.) was used for the capacitance and the EIS measurements in the glove box. The frequency range was from 50 mHz to 1 MHz with 10 mV AC electrical perturbation. The EIS measurements were performed under illumination at 100 mW/cm^2^ and at various voltages. Absorption spectra were recorded with a UV–vis absorption spectrophotometer (Perkin Elmer Lambda 950). The photoluminescence (PL) and time-resolved photoluminescence (TRPL) spectra of annealed triple-cation perovskite films (control and treated with 2I-Ac, 2Br-Ac, and 2Cl-Ac molecules) on glass were recorded with Edinburgh Instruments. The samples were excited by a pulsed 655 nm wavelength laser (EPL-655, pulse width: 59.1 ps, Edinburg Instruments Ltd.). PL measurements were conducted with monochromatic light as an optical excitation source using a 450 W Xe arc lamp. X-ray photoelectron spectroscopy (XPS) was carried out using a Thermo Scientific K-Alpha spectrometer equipped with a monochromatic and focused (spot dimensions 300 by 300 μm) Al Kα radiation (1486.68 eV). ^1^H and ^13^C nuclear magnetic resonance spectra of solutions (NMR) in DMSO-*d*_6_ were acquired on a MERCURYplus-AS 400 at 400 MHz at room temperature.

## Supporting Information Available

The film thicknesses of the bare and modified triple-cation perovskite; the PL of the substrate glass/triple-cation perovskite and glass/triple-cation perovskite/HTMs; ^1^H NMR spectra of the 2Br-Ac and 2Br-Ac + PbI_2_ solution in DMSO-*d*_6_; the XPS full survey spectra and high-resolution XPS spectra of Br 3d of the triple-cation perovskite thin films; the AFM topographic images of the triple-cation perovskite thin films; and the photograph images of the CB + 2Cl-Ac solution and the triple-cation perovskite solar cell fabricated with the CB + 2Cl-Ac solution (PDF)
