# Supplementary material for: Simultaneous Optimization of Charge Transport Properties in a Triple-Cation Perovskite Layer and Triple-Cation Perovskite/Spiro-OMeTAD Interface by Dual Passivation
Source: ACS Omega. 2022 May 17;7(21):17907–20. doi: 10.1021/acsomega.2c01195 (PMC9161386; doi:10.1021/acsomega.2c01195)
Supplement: Supplementary file 1 — ao2c01195_si_001.pdf [file ao2c01195_si_001.pdf]

Supporting Information

Simultaneous Optimization of Charge Transport Properties in Triple-cation Perovskite Layer and Triple-cation Perovskite/Spiro-OMeTAD Interface by Dual Passivation

Adem Mutlu[1], Tamer Yeşil[1], Deniz Kıymaz[1], Ceylan Zafer[1*]

[1]*Solar Energy Institute, Ege University, 35100, Izmir, Turkey*

Corresponding authors

C. Zafer. E-mail: ceylan.zafer@ege.edu.tr

Tel:+90 232 3111245

This file includes:

Supplementary Figures S1 to S10

Supplementary Tables S1 and S2

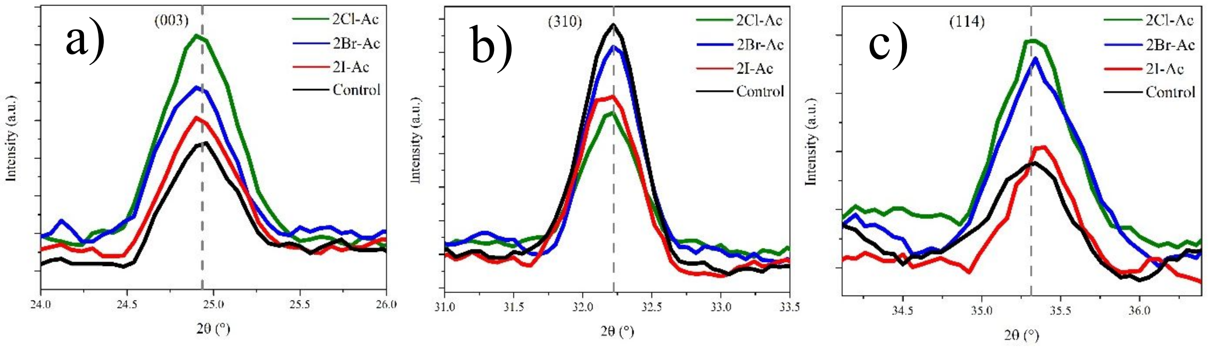

Figure S1. Xrd plots of a) 2θ=24.5°, b) 2θ=31.8° and c) 2θ=35.3° peaks

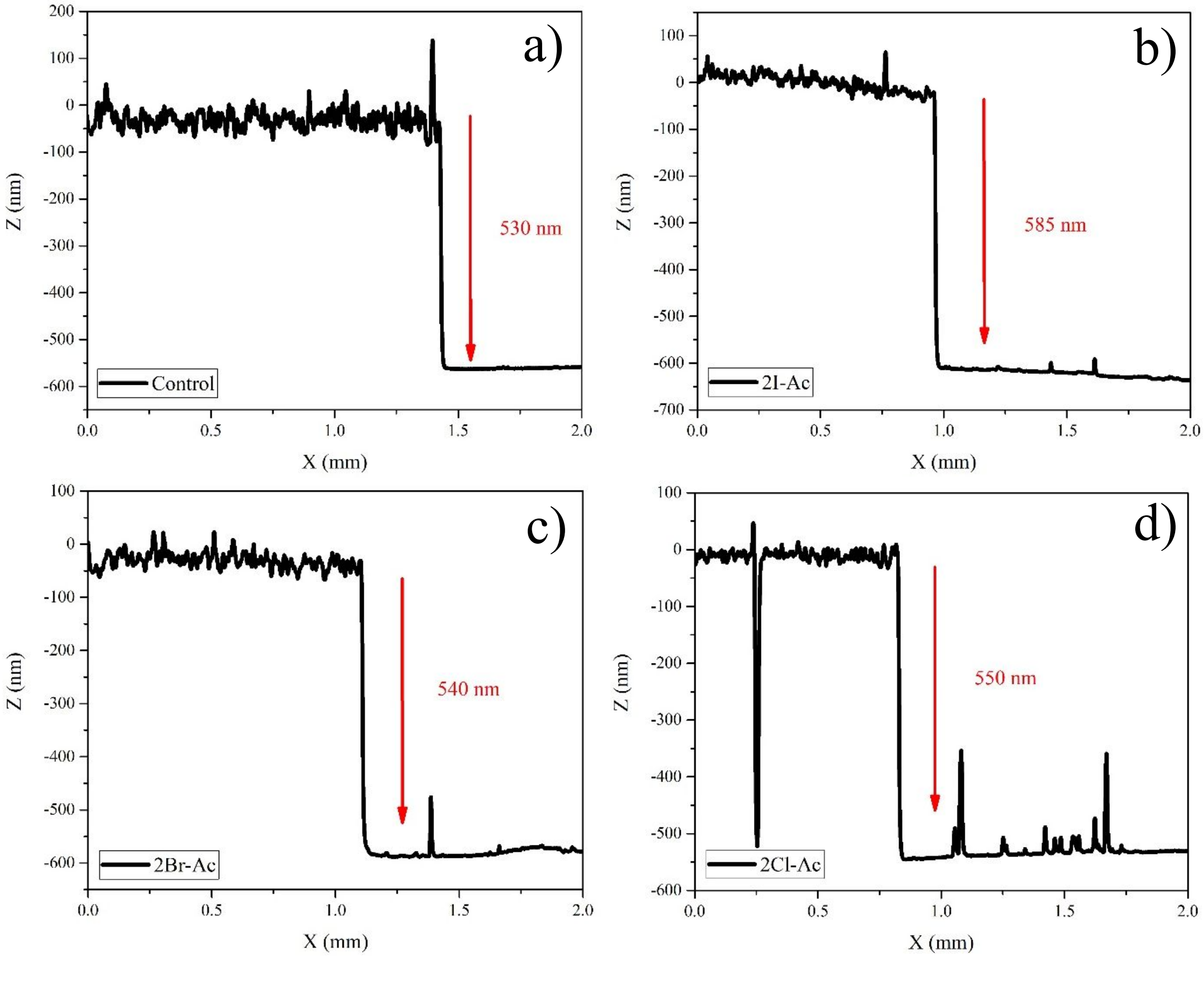

Figure S2. The film thicknesses of the a) control, b) 2I-Ac, c) 2Br-Ac and d) 2Cl-Ac modified triple-cation perovskite

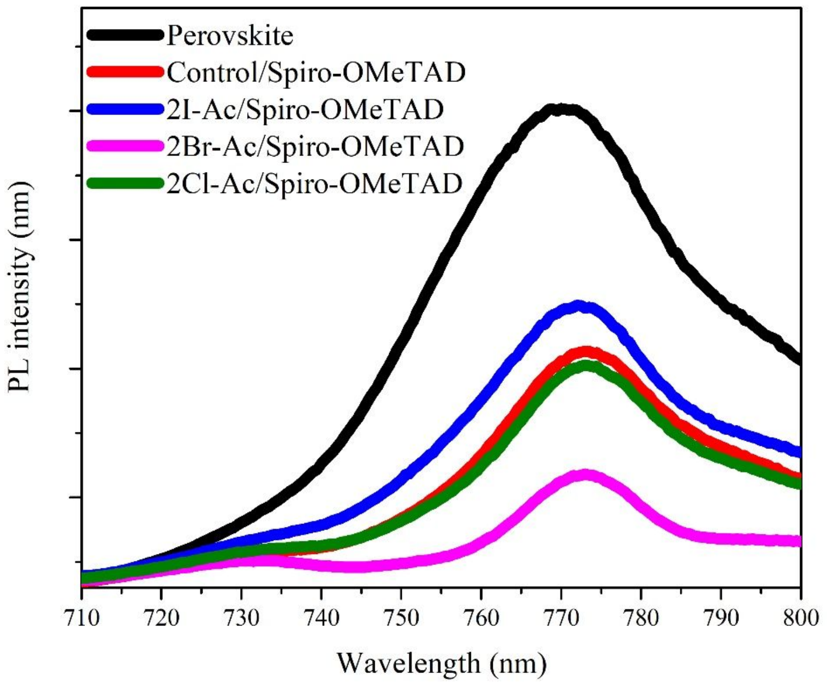

Figure S3. The PL of the substrate glass/perovskite and glass/perovskite/HTMs

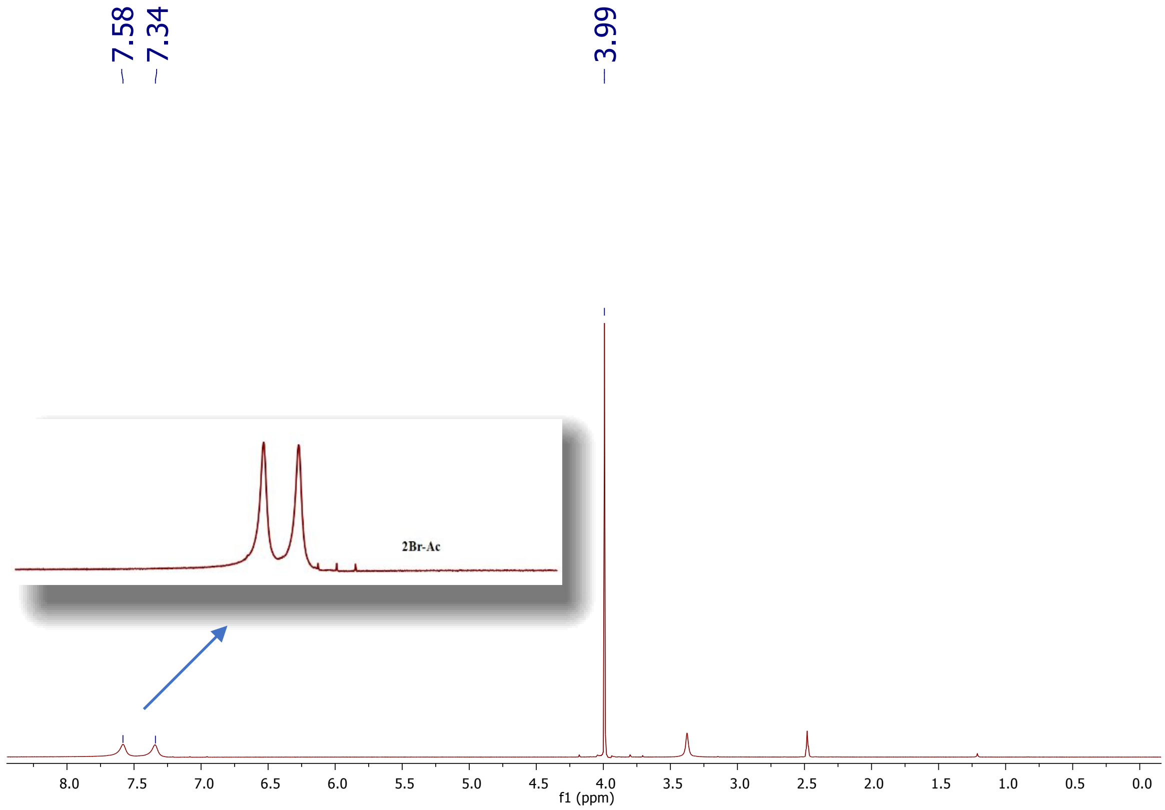

Figure S4. ^{1}H-NMR spectrum of 2Br-Ac solution in DMSO-d_6

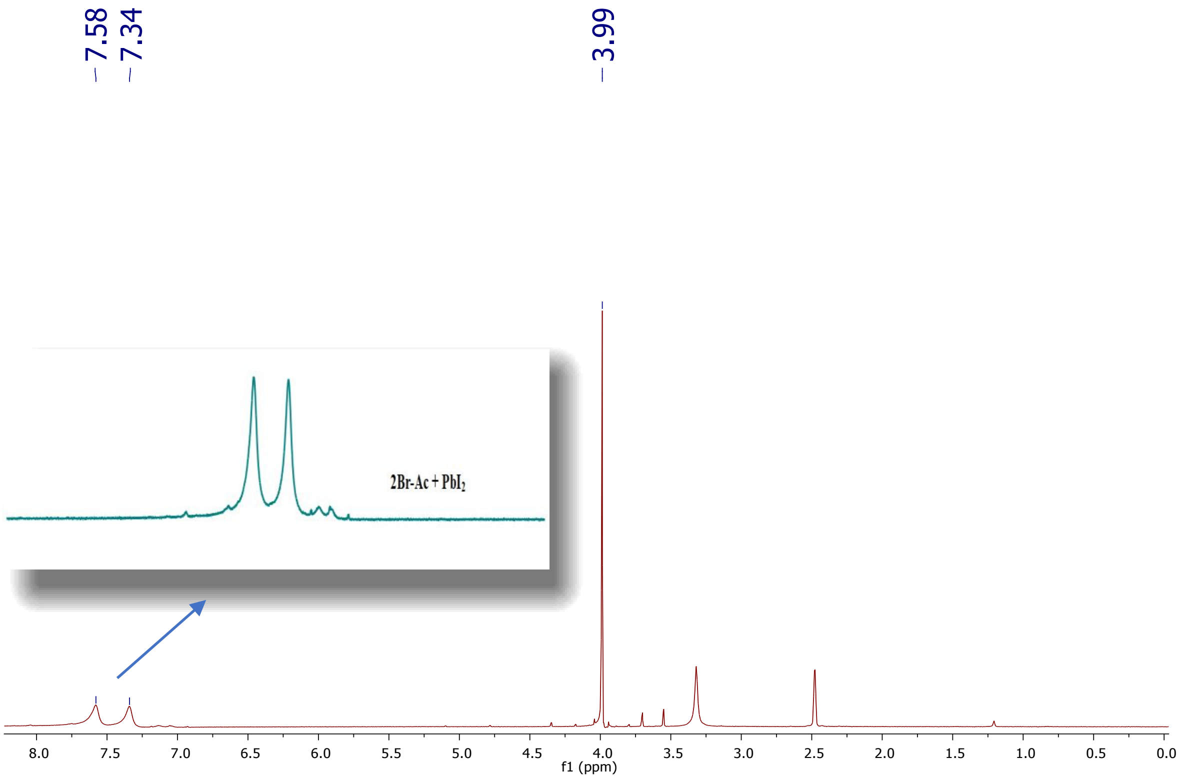

Figure S5. ^{1}H-NMR spectrum of 2Br-Ac + PbI_2 solution in DMSO-d_6

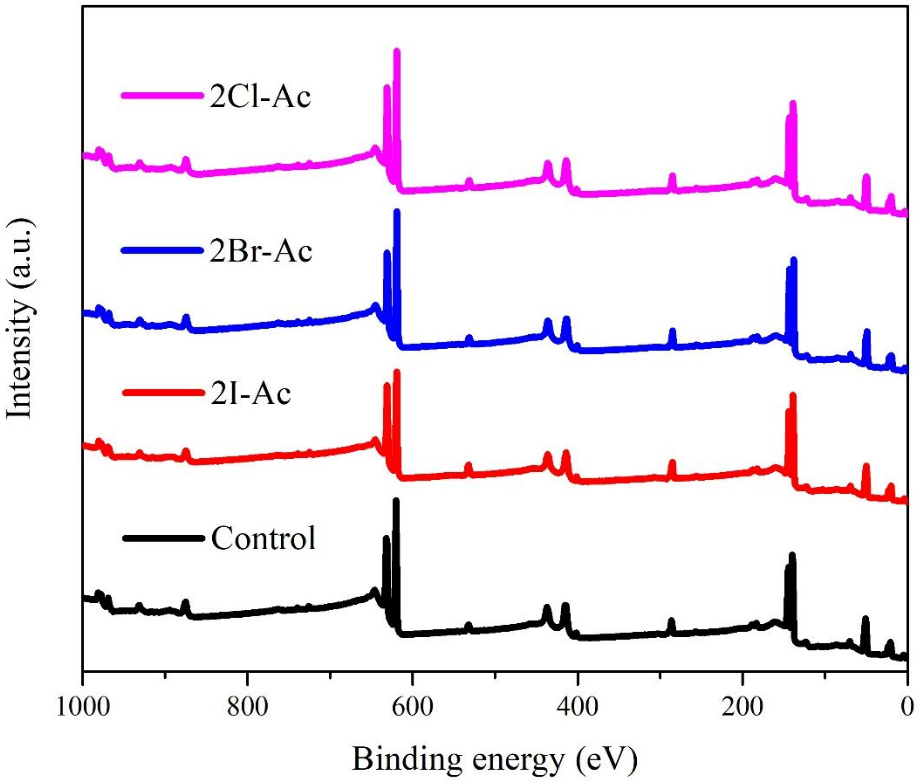

Figure S6. The XPS full survey spectra of perovskite thin films washed with CB, 2I-Ac, 2Br-Ac, and 2Cl-Ac molecules.

Table S1. Br 3d3/2 and 5/2 high resolution spectra of triple-cation perovskite thin films without and with 2I-Ac, 2Br-Ac and 2Cl-Ac molecules.

Anti-solvents	Br $3d_{3/2}$ (eV)	Br $3d_{5/2}$ (eV)
**CB**	70.82	69.83
**2I-Ac**	69.12	68.12
**2Br-Ac**	69.00	68.05
**2Cl-Ac**	69.17	68.22

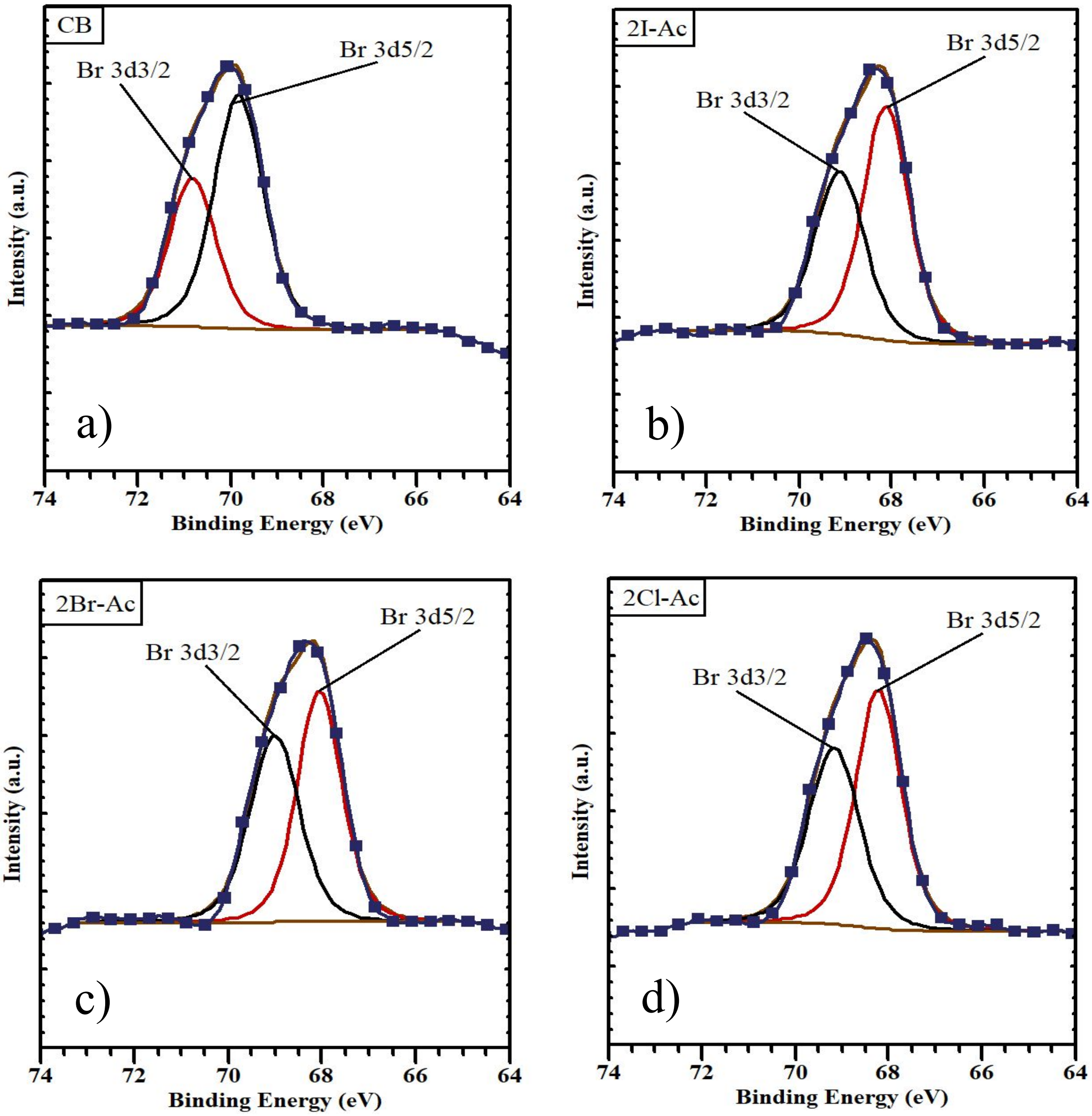

Figure S7. Br 3d spectra of triple-cation perovskite thin films a) control, b) 2I-Ac, c) 2Br-Ac and d) 2Cl-Ac molecules.

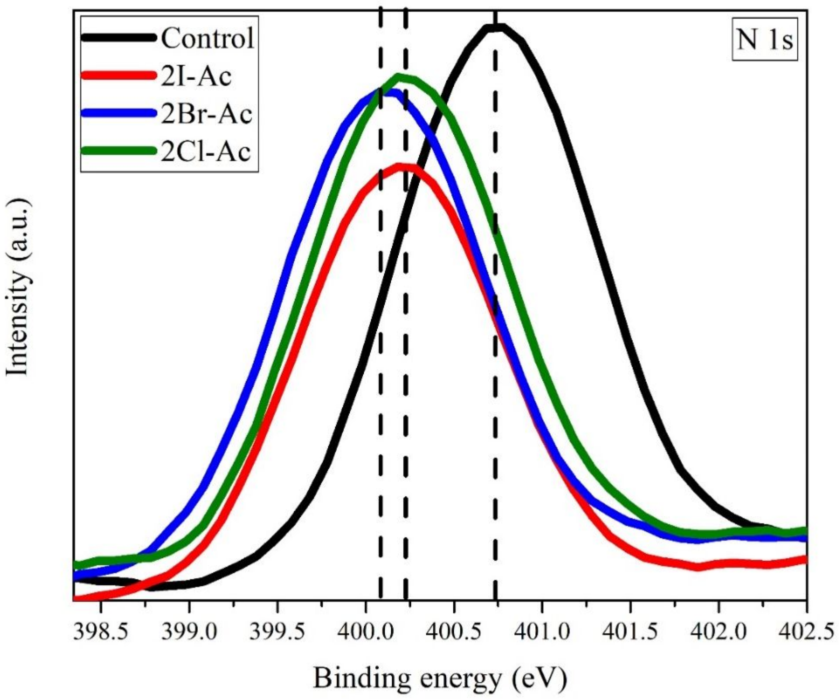

Figure S8. XPS spectra N 1s for the triple cation perovskite films without and with 2I-Ac, 2Br-Ac and 2Cl-Ac molecule modification.

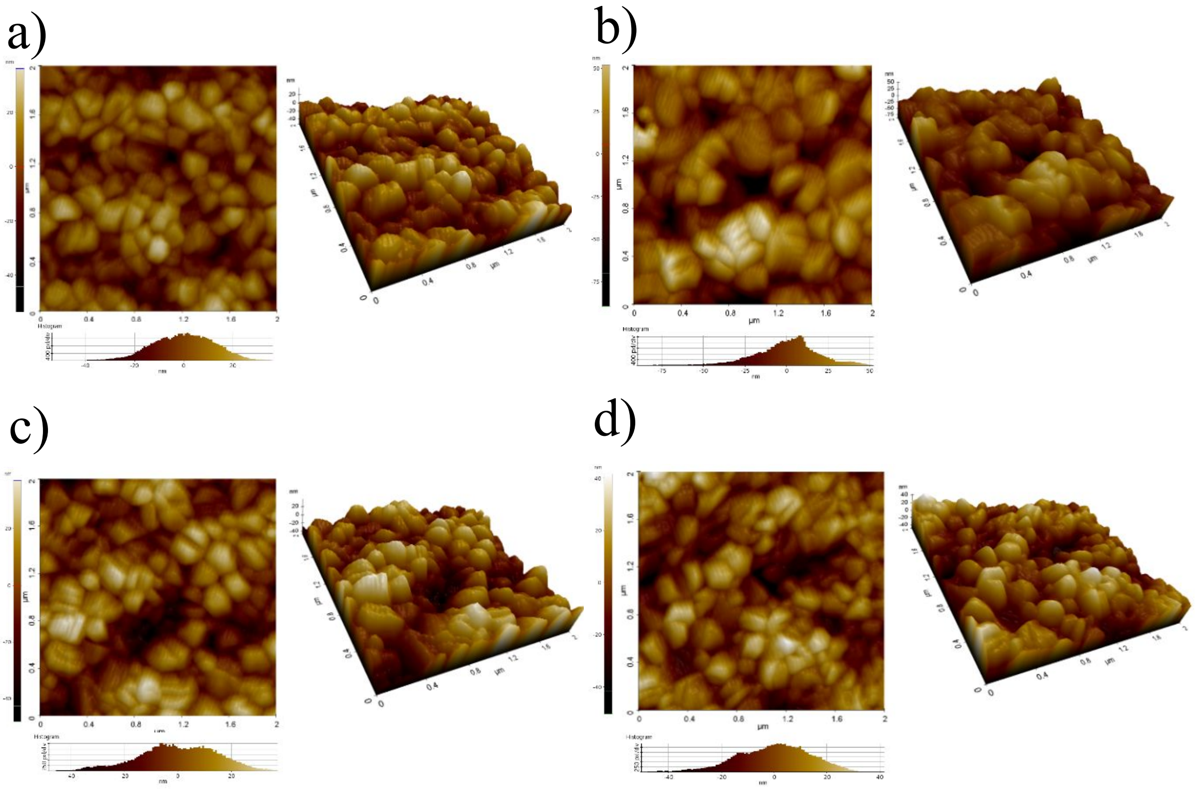

Figure S9. Surface morphology and 3D images of perovskite films of $2 \times 2 \mu m^2$, (a) control, (b) 2I-Ac, (c) 2Br-Ac and (d) 2Cl-Ac molecules.

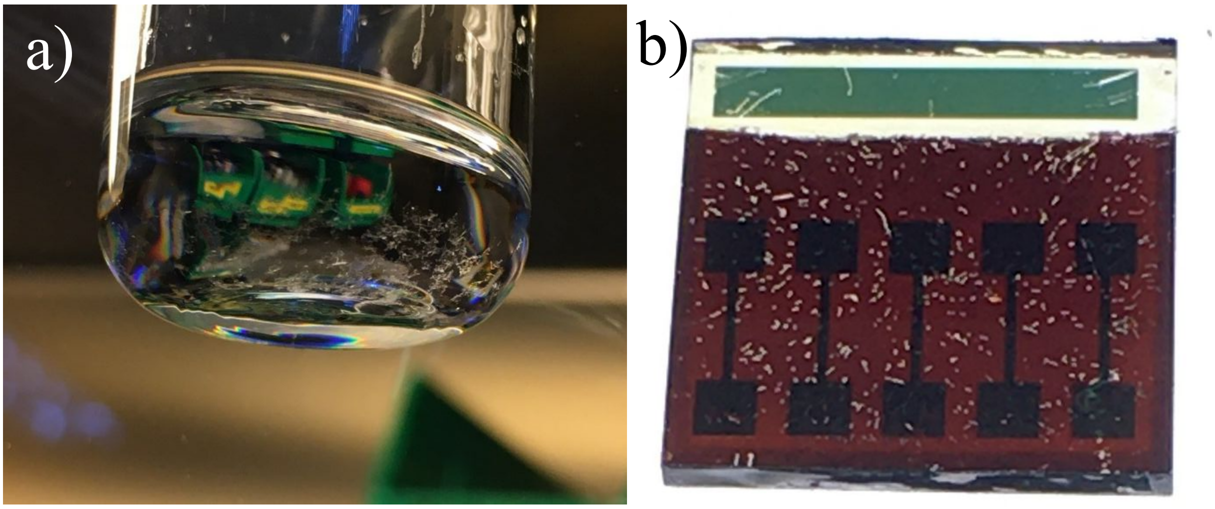

Figure S10. a) The photograph of CB + 2Cl-Ac (1ml/1mg) solution, b) photograph image of perovskite solar cell fabricated with CB+2Cl-Ac. *(The photographs were taken by the authors in the laboratory.)*

Table S2. Best and average photovoltaic performance of the PCSs prepared with CB, 2I-Ac, 2Br-Ac, and 2Cl-Ac.

	J_{SC}^{J-V} (mA/cm^2)	J_{SC}^{IPCE} (mA/cm^2)	Error $_{(Jsc)}$ (%)	V_{oc} (mV)	FF (%)	PCE (%)	Avg. PCE (%)
**Control**	21.6	18.8	13.0	1112	73.3	17.6	17.0
**2I-Ac**	21.3	18.4	13.6	1112	69.7	16.5	15.6
**2Br-Ac**	24.4	19.2	21.0	1112	71.9	19.5	19.0
**2Cl-Ac**	22.6	19.0	15.9	1075	66.3	16.1	15.5

Data were reversely scanned. Data averaged from 10 individual devices.
